# The Diagnostic Value of Nuclear Matrix Proteins in Bladder Cancer in the Aspect of Environmental Risk from Carcinogens

**DOI:** 10.1155/2017/9643139

**Published:** 2017-08-08

**Authors:** Beata Szymańska, Ewa Sawicka, Anna Guzik, Romuald Zdrojowy, Anna Długosz

**Affiliations:** ^1^Department of Toxicology, Faculty of Pharmacy, Wrocław Medical University, Wrocław, Poland; ^2^Department of Urology and Urological Oncology, Wrocław University Hospital, Wrocław, Poland

## Abstract

**Background:**

The interaction of environmental factors with genetic susceptibility and detoxification level seems to be an important causative factor in bladder cancer (BC). The aim of this study was to look for a BC marker panel which reflects the environmental risk. The nuclear matrix protein 22 (NMP22), bladder cancer-4 (BLCA-4), and total level proteins NMP22 and BLCA-4 (NMBL) in BC patients with genetic predisposition NAT2 (classified as slow acetylators, SA), DNA damage (8-OHdG), and detoxification by isoenzyme GST*π* activity were measured.

**Materials and Methods:**

The urine and blood from 91 BC patients and controls were examined, also according to tumor stage (T) and grade (G). The participants completed a questionnaire in order to evaluate environmental risk.

**Results:**

Most patients (75.3%) were previous or actual smokers. The levels of 8-OHdG, NMP22, BLCA-4, NMBL, and GST*π* were significantly higher in BC (*p* ≤ 0.001). The majority of patients (59.3%) were slow acetylators (SA). The highest BLCA-4/8-OHdG correlation was observed in total BC and SA smokers.

**Conclusions:**

The total pool of nuclear matrix proteins in the urine (NMBL) has a higher diagnostic value in bladder cancer than single proteins. The particular value of BLCA-4 and GST*π* in the aspect of environmental risk was noted.

## 1. Background

Many xenobiotics are able to induce urinary bladder cancer (BC) [1–4]. One of the cancerogenic mechanisms is based on the release of bladder toxic carbocations from xenobiotic conjugates, by hydrolysis, during its elimination with urine. This occurs, for example, during exposure to aromatic amines. The formation of toxic electrophiles is particularly intensive in slow acetylators (SA), with NAT2 genotype. The SA are more susceptible to BC during exposure to some xenobiotics [5, 6].

The diagnostic problems of BC (at present, the main diagnostic and prognostic tool is cystoscopy), as well as the role of environmental exposure (about 50% of BC patients are smokers and smoking is considered as the primary cause of BC development) and genetic predisposition (genotype of N-acetyl-transferase NAT2 and glutathione transferase GST), have motivated us to look for BC markers which combine the risk factors mentioned above and are less invasive than cystoscopy with biopsy.

The aim of the study was to evaluate the diagnostic value of nuclear matrix protein 22 (NMP22) and bladder-specific proteins (BLCA-4) in BC patients with genetic predisposition (NAT2 genotype) and with DNA damage measured by 8-hydroxy-2′-deoxyguanosine (8-OHdG) level, as well as detoxification by isoenzyme GST*π* level in the urine. We assumed that it is possible to find a BC marker panel which also reflects the environmental risk. The evaluation of NMP22, BLCA-4, and the joint (total) NMBL (NMP22 + BLCA-4) level in BC patients with the slow acetylation genotype (SA) and increased 8-OHdG level has not previously been examined but seems to be an interesting attempt to consider the environmental influence in BC diagnosis.

Bladder cancer incidence is strongly related to age, with the highest incidence rates being in older humans. The median age at diagnosis is 69 years in men and 71 in women. The BC incidences increase twice in people aged over 85. In the UK, in 2012–2014, more than half of the BC cases were diagnosed in people aged 75 and over [7, 8].

Nuclear matrix proteins are known to be connected with the cell's genetic expression and response to various xenobiotics stimuli. Their functions include DNA organization, stabilization, and orientation during replication, determination of nuclear morphology, organization of gene regulatory complexes, and synthesis of RNA [9]. Among the various nuclear matrix proteins, NMP22 and BLCA-4 are especially of interest in bladder cancer. They are involved in genome fragmentation and cellular replication. It seemed interesting to characterize the sensitivity of these markers in NAT2 genotype BC patients [10].

NMP22 is a nuclear matrix protein located in the mitotic spindle. It is involved in microtubule assembly and in fragmentation of the genome into new G1 nuclei during cellular replication. The NMP22 is released from cells during apoptosis. Its level is higher in bladder cancer cells compared to normal ones. The NMP22 concentration increases in the urine of BC patients and is rated as a highly sensitive but poorly specific marker [11–13].

BLCA-4 is currently under evaluation as a tumor-specific BC marker. The expression of this protein is in the very early stage of illness. BLCA-4 protein is expected to be an early noninvasive diagnostic marker of BC, because it appears in the urine even before tumor formation. The mechanism of BLCA-4 is poorly understood. Investigations did not show any proangiogenic activity [14–16].

The risk of exposure to cancerogenic xenobiotics is closely connected with detoxification abilities of the organism, especially with glutathione transferase (GST) levels [17, 18]. GSTs are metabolizing enzymes of phase II, which protect normal cells by detoxification of such carcinogens like polycyclic aromatic hydrocarbons (PAH) by glutathione conjugation. Except for the NAT2 genotype, also GSTM1 null genotype increased the risk of BC [19]. The isoenzyme GST*π* is particularly involved in cancerogenesis [20]. Our previous investigation showed that GST*π* can be useful in BC diagnosis [21]. A doubling of the expression of GST*π* in the urothelium of BC patients was reported [22].

N-Acetyltransferases (NATs) are enzymes that reduce the toxicity of compounds with an amino group by catalyzing its acetylation. The NAT2 polymorphism leads to three genotypes of acetylation, including slow acetylators, more sensitive at risk of bladder cancer in exposure to aromatic amines. Aromatic amines are permanently present in the environment (car pollution, tobacco smoke, and combustion products) and may also accumulate in the fatty tissue [5, 23, 24].

DNA damage, including oxidative DNA changes caused by free radicals, plays an important role in the pathogenesis of cancer. One of the products of DNA oxidative damage is 8-hydroxy-2′-deoxyguanosine, an 8-oxoguanine metabolite. There is evidence that 8-OHdG can be an indicator of genetic damage upon exposure to xenobiotics. It has been detected as the most frequent base modification in DNA. It leads to GC-TA transformation most frequently found in the ras oncogene [25–28].

Hence, it seems that the analysis of the correlation between nuclear matrix protein and markers of DNA oxidative damage as well as the detoxification ability could bring about important information in the diagnosis of BC, which in the examined markers could reflect environmental exposure to carcinogens. This is a new trial in research of BC markers which takes into consideration genetic susceptibility and environmental carcinogens.

## 2. Materials and Methods

The study was conducted in a group of 91 patients with bladder cancer (BC) who were hospitalized at the Department and Clinic of Urology and Urological Oncology at Wroclaw Medical University in 2013–2015. The group included 74 men (81.13%) (age range 39–82 years) and 17 women (18.9%) (age range 60–87 years). The mean age of patients was 68 ± 9.5 years.

The control group (C) included 25 healthy volunteers: 18 men (72%) and 7 women (28%) aged 54–81 years (mean age 65 ± 6.9) without any urinary tract diseases. The B and C groups have of similar socioeconomic status. All the patients as well as volunteers were free of drug and alcohol.

BC patients were divided into subgroups depending on tumor stage T and tumor grade G according to TNM Classifications of Malignant Tumours [29]. The division was based on the histopathological examination and was conducted by the Department of Pathology, Wrocław Medical University. The subgroups were Ta (43 cases, 45.3%), T1 (31 cases, 34.1%), T2 (10 cases, 11%), TIS (7 cases, 7.7%), G1 (40 cases, 47.6%), G2 (41 cases, 48.8%), and G3 (3 cases, 3.6%) (Table 1).

The nuclear matrix proteins NMP22, BLCA-4, isoenzyme GST*π*, and 8-OHdG were measured in the urine of patients with BC and the control group (C) using the same method, but NAT2 was measured in the blood. Determination of NMP22 and GST*π* isoenzyme was performed in 91 BC patients, while determination of BLCA-4 and 8-OHdG was performed in 63 patients. The material, midstream morning urine samples (10 mL), was collected in polystyrene containers (Aptaca, Italy). At first, the urine samples were centrifuged for 10 minutes (1438 ×g at 4°C). The obtained supernatant was transferred to Eppendorf tubes and stored at −80°C for further investigation. Marker levels were detected in the urine using an enzyme-linked immunosorbent assay (ELISA) according to the manufacturer's instructions (Shanghai Sunred Biological Technology NMP22 Kit; Cusabio Biotech BLCA-4; Human Pi GST EIA-EKF Diagnostics; Human 8-OHdG Check ELISA JaJCA Japan Institute for the Control of Aging). The obtained marker values were calculated in relation to the urine creatinine level previously estimated by Jaffe's routine method. Under alkaline conditions, creatinine reacts directly with picric ions to form a reddish complex, the absorbance of which was measured at *λ* = 520 nm. The volume of urine samples in each test was 100 *µ*L. NAT2 genotype was determined according to a method described in the literature [30–32]. Blood samples for NAT2 assessment were collected from patients into plastic tubes (BD Vacutainer, USA), with an anticoagulant (buffered sodium citrate). The blood was kept at −80°C until use. Acetylation of NAT2 genotype was tested in blood of patients (*n* = 91) using the polymerase chain reaction technique (PCR-Plus Master Mix, A&A Biotechnology). DNA was isolated from whole blood using a Blood Mini Kit (A&A Biotechnology, Poland), according to the manufacturer's protocol supplied with the kit reagents. The purity and quality of DNA were controlled by electrophoresis on agarose gel. Amplification of the NAT2 gene fragment was carried out using a MixPlus 2x PCR Kit (A&A Biotechnology, Poland). Primers used in the reaction had the sequences NAT2 F 5′ GCT AGC GGG GGA TCC TCT TC 3′ and NAT2 R 5′ TTG GAT GGT TAC ACA ACA AGG G 3′. Reactions were conducted according to the following program: 94°C, 4 minutes, 34 cycles [94°C, 30 seconds; 59°C, 30 seconds; 72°C, 45 seconds]; 72°C, 5 minutes; 8°C, 10 seconds. After amplification, the NAT2 gene fragment was digested with restrictive enzymes at the site of potential mutations (Enzymes Fast Digest of Thermo Scientific: KpnI, DdeI, BamHI, TaqI). The results were evaluated after electrophoresis on agarose gel under UV light (Transilluminator microDOC, Cleaver Scientific Ltd.).

From the whole group of 91 patients with BC, 85 patients completed a questionnaire in order to assess the impact of environmental factors (smoking, residence, occupation, and exposure to chemicals) on the incidence of BC. They also answered the question concerning drug and alcohol consumption. The same questionnaire was given to the control group.

The project received the permission of the Bioethics Committee of Wrocław Medical University (number KB-13/2014 and number KB-276/2016), and all patients provided signed written informed consent. The study was conducted in accordance with the Declaration of Helsinki (1964), and all participants provided signed written consent.

### 2.1. Statistical Analysis

Statistical analysis was conducted with Statistica PL software (version 12.0). The normality of distribution was checked with the Lilliefors test. Student's *t*-test for parametric data and the Mann–Whitney* U* test for nonparametric data were used for variables. Nonparametric tests (Wald-Wolfowitz, Kolmogorov-Smirnov, and Mann–Whitney* U*) were applied. The results of all three tests were similar, so Mann–Whitney* U* test was chosen. Values of *p* < 0.05 were considered as statistically significant. The associations between continuous variables were analyzed by Spearman for nonparametric data and Pearson for parametric data. Also, sensitivity, specificity, accuracy (ACC) of the method, positive predictive value (PPV), negative predictive value (NPV), positive likelihood ratio (LR+), negative likelihood ratio (LR−), and odds ratio (OR) were determined. For the purpose of the analysis, the individual characteristics of patients, gender, age, smoking, pathological stage (Ta, T1, T2, or TIS), and grade (G1, G2, or G3), were correlated with estimated biochemical markers.

The receiver operating characteristic (ROC) curves were estimated according to Youden. The area under the curve (AUC) and best cut-off point were calculated employing ROC analysis which evaluated the relation between sensitivity and specificity of markers according to StatSoft [33]. The creation of a multifactor model was conducted using progressive stepwise regression, and to evaluate the model the Wald test and the Hosmer test were used.

## 3. Results

### 3.1. Questionnaire

The questionnaire completed by 85 BC patients indicated that the majority of patients were former or current smokers (75.4%, 64 patients; nonsmokers 24.7%, 21 patients); 58 patients (71%) lived in a city; 21 patients (34.1%) had contact with pesticides; 13.4% confirmed exposure to chemicals (living near factories). Observed results are presented in Table 2.

### 3.2. Nuclear Matrix Proteins

The mean concentration of protein NMP22 in the urine of patients (BC) was 10.846 ng/mg cr. while in the control group (C) it was 5.147 ng/mg cr. and was significantly higher in BC patients (*p* ≤ 0.001). Similarly, the concentration of BLCA-4 (BC group, 3.015 ng/mg cr.; C group, 0.878 ng/mg cr.) (*p* ≤ 0.001) and the sum of both proteins (NMBL) (*p* ≤ 0.001) were significantly higher in BC (Table 3). There were no significant differences in examined protein concentrations between BC men and women or between BC smokers and nonsmokers (Table 3).

**Figure 1:**
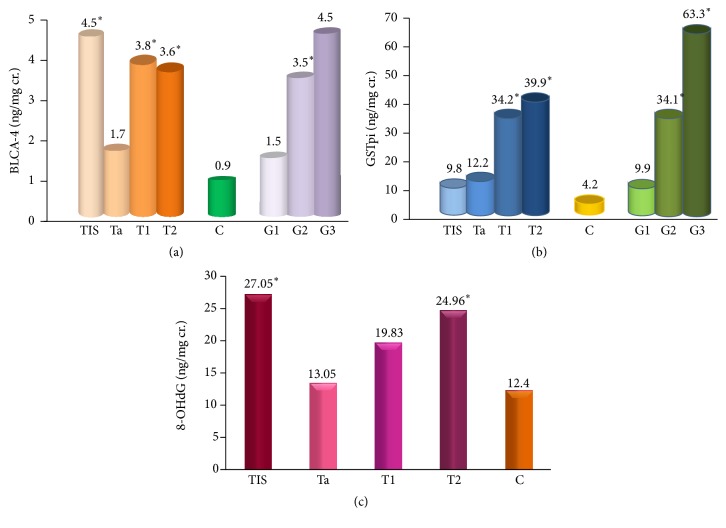
(a) BLCA-4, (b) GST*π*, and (c) 8-OHdG in tumor stage (T), in tumor grade (G), and in control group (C). (a) *∗*: statistically significant difference between subtypes Ta/T1, Ta/T2, Ta/TIS; G1/G2. (b) *∗*: statistically significant differences between subtypes Ta/T1, Ta/T2, TIS/T1, TIS/T2; G1/G2, G1/G3. (c) *∗*: statistically significant differences between subtypes Ta/TIS, Ta/T2.

The analysis of NMP22, BLCA, and NMBL proteins performed according to tumor stage (T) and grade (G) [Ta (*n* = 43), T1 (*n* = 31), T2 (*n* = 10), and TIS (*n* = 7); G1 (*n* = 40), G2 (*n* = 41), and G3 (*n* = 3)] showed no differences between subgroups T and G in NMP22 level; however, there were statistically significant differences in BLCA-4 concentration between Ta and T1 (*p* = 0.0115), Ta and T2 (*p* = 0.0288), and Ta and TIS (*p* = 0.0255) (Figure 1(a)). The BLCA-4 level also increased with tumor grade, showing a statistically significant difference between G1 and G2 (*p* = 0.0008) (Figure 1(a)). The differences were also noted for NMBL between TIS and Ta (20.31/11.08 ng/mg cr., *p* = 0.03) and also between G1 and G2 (10.87/14.69 ng/mg cr., *p* = 0.008). A positive correlation (*R* = 0.295; *p* = 0.019) between the level of NMBL and local progression of tumor stage T was noted. In order to reach the final conclusion about T and G analysis, the experiments should be continued with a larger number of cases.

### 3.3. Other Parameters

The mean concentration of isoenzyme GST*π* in the examined group (BC) was 22.545 ng/mg cr. while in the control (C) it was 4.210 ng/mg cr. and was statistically higher in patients with bladder cancer (*p* ≤ 0.001). There was no statistically significant difference in GST*π* in the BC group of women and men, or between BC smokers and nonsmokers (Table 3). A positive correlation of GST*π* with tumor stage (T) (*R* = 0.4062; *p* ≤ 0.001) and tumor grade (G) (*R* = 0.4206; *p* ≤ 0.001) was found. No correlation was observed between GST*π* and age, sex, or smoking. Statistically significant differences were found between tumor stages T: Ta and T1 (*p* = 0.0004), Ta and T2 (*p* = 0.0027), T1 and TIS (*p* = 0.0289), and T2 and TIS (*p* = 0.0358). The highest GST*π* level was demonstrated in T2 (39.901 ng/mg cr.), the most invasive one from examined tumors. GST*π* concentration increased with the tumor stage T. Especially important is the difference between invasive and noninvasive stages (e.g., Ta and T2, *p* = 0.003) and tumor grade: G1 and G2 (*p* ≤ 0.001) and G1 and G3 (*p* = 0.0207) (Figure 1(b)).

The concentration of 8-OHdG was slightly but significantly higher in patients (BC) (18.914 ng/mg cr.) than in controls (C) (12.393 ng/mg cr.) (*p* = 0.0456) (Table 3) and in smoking patients (21.486 ng/mg cr.) in comparison to the BC nonsmokers (14.34 ng/mg cr.) (*p* = 0.0353). A positive correlation between the 8-OHdG excreted into urine and the habit of cigarette smoking in patients (*R* = 0.3826; *p* = 0.003) and the tumor stage (T) (*R* = 0.3493; *p* = 0.005) was noted. No correlation was observed between 8-OHdG and sex, age, or tumor grade (G).

The dependence between the concentration of 8-OHdG and invasiveness of cancer T was estimated. The highest concentration of 8-OHdG was observed in TIS (27.045 ng/mg cr.) and T2 (24.959 ng/mg cr.), the lowest in Ta tumor (13.045 ng/mg cr.). It could be caused by characteristic of TIS (high-grade carcinoma with a flat nonpapillary configuration) and T2 (highly invasive) which results in higher DNA damage level at TIS. The significance of the difference (*p* = 0,02) was not high, so experiments should be repeated in a larger population to get the final conclusion. Between Ta and TIS (*p* = 0.0198) and also between Ta and T2 (*p* = 0.025), statistically significant differences in the concentration of 8-OHdG were observed (Figure 1(c)). No such differences were found by analyzing the degree of tumor grade.

A correlation of 8-OHdG with nuclear matrix protein NMP22 (*R* = 0.4323; *p* = 0.0037), BLCA-4 (*R* = 0.4876; *p* = 0.0009), and NMBL (*R* = 0.4894; *p* = 0.0008) was obtained in the group of smokers. In nonsmoking patients, no correlations were observed. The result suggests that the level of 8-OHdG reflects the genotoxic effects of environmental toxins (smoking).

### 3.4. Genotype of NAT2 Acetylation in Patients with Bladder Cancer

The conducted analysis of the NAT2 genotype in the examined group (BC) showed three types of acetylation: slow (SA), intermediate (IA), and fast (FA). The presence of both wild-type alleles (NAT2^*∗*^4) determines the fast acetylation. Intermediate acetylators are heterozygotes, having NAT2^*∗*^4 on one allele, while the other allele is mutated. People with two mutated alleles of the NAT2 gene are classified as slow acetylators.

In the group of patients (BC), the slow type of acetylation dominated (SA, 54 patients, 59.3%); 29 patients (31.9%) were classified as intermediate acetylators (IA) while 8 patients (8.8%) were classified as fast acetylators (FA). Quantitative and percentage distribution of acetylation genotypes is presented in Table 4. The majority of patients (59,3%) had the slow acetylation genotype of NAT2 (SA).

The values of determined parameters depending on the status of NAT2 acetylation are shown in Table 5. There were no statistically significant differences between groups.

The positive correlations between parameters in the group of slow acetylators (SA) are shown in Table 6.

The 8-OHdG showed positive correlations with all parameters, NMP22, BLCA-4, NMBL, and GST*π*, and with the habit of smoking. A highly significant correlation was observed between nuclear matrix protein level and 8-OHdG, highest in SA for BLCA-4 (*R* = 0.583, *p* = 0.0001) (Table 6). Also, analysis of BC-SA smokers showed the highest correlation value with 8-OHdG for BLCA-4 (*R* = 0.6942; *p* = 0.0001) (Table 6). These show that, among all examined nuclear matrix proteins, BLCA-4 is the best marker which reflects the environmental risk of chemical cancerogenesis. In the SA BC group, the BLCA-4, NMBL, GST*π*, and 8-OHdG correlated with stage of cancer but with tumor grade (G) only GST*π*. No correlation between markers and age or sex was detected in the SA group.

### 3.5. Statistical Evaluation of Diagnostic Value of Tested Parameters in BC

The diagnostic value of the tested parameters was evaluated for the whole group of patients (BC) and the group of slow acetylators (SA). The results are summarized in Table 7.

The highest specificity was observed for NMP22 (96%), NMBL (96%), and 8-OHdG (100%). 8-OHdG was characterized by low sensitivity but the highest specificity (100%).

**Figure 2:**
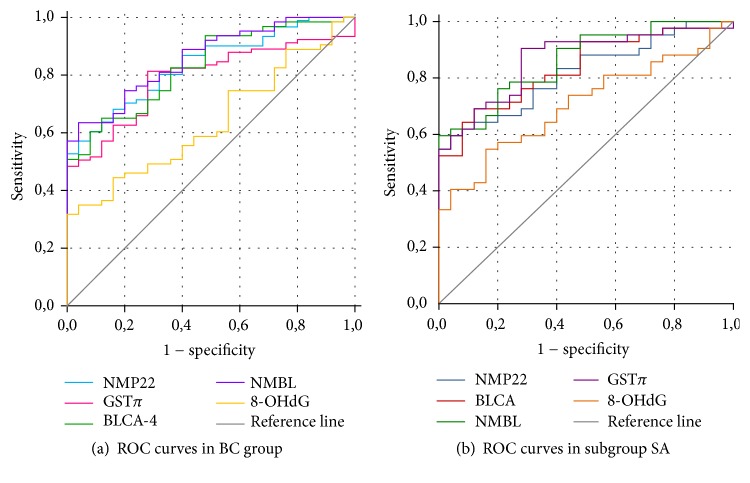
Receiving operating characteristic (ROC) analysis of NMP22, BLCA-4, NMBL, GST*π*, and 8-OHdG level in whole BC group (a) and SA subgroup (b).

Higher sensitivity for NMP22 and BLCA-4 was observed in the SA group, especially for GST*π* and 8-OHdG. GST*π* in the BC group showed the highest sensitivity (81%) and accuracy (ACC) (79%). The comparison between the whole BC and SA BC showed an increase in specificity for NMBL (96% for BC, 100% for SA). The ACC factor of all parameters for the SA group was higher than for the whole group of patients (BC). NMBL scored the most effective prediction value in the SA group with PPV increasing to 100%. From the conducted analysis, it was found that the sensitivity of GST*π* and 8-OHdG tests was significantly higher in the SA group, as were factor ACC and the diagnostic value of tests. For GST*π* (AUC = 0.866), the status of a good diagnostic value in the SA group was demonstrated. AUC for GST*π* has a good diagnostic value for slow acetylators (0.866), higher than for the whole BC group (Figure 2).

ROC areas under the curve for NMP22, BLCA-4, NMBL, and GST*π* were similar but only for matrix proteins did they reach a level that can be considered as a good diagnostic indicator [0.8–0.95]: NMP22, 0.836; BLCA-4, 0.830; NMBL, 0.858. These ROC curves show the relation between sensitivity and specificity and are able to indicate optimal values of parameters (cut-off points, Youden diagnosis). High (100%) specificity and 100% PPV in the BC group as well as the above-mentioned correlations with 8-OHdG show the utility of nuclear matrix proteins and GST*π* in terms of environmental risk in the diagnosis of bladder cancer. The statistical Hosmer pointed at three parameters: GST*π* [OR (95% CI) = 11.18 (1.92–65.25), *p* = 0.0073], NMBL [OR (95% CI) = 58.88 (4.53–764.66), *p* = 0.0018], and smoking [OR (95% CI) = 36.23 (5.57–235.64), *p* = 0.0002].

## 4. Discussion

Nuclear matrix is an active cellular environment where DNA replication and RNA synthesis take place. Changes in nuclear structure can affect expression of genes which play an important role in carcinogenesis. Nuclear matrix proteins (NMPs) bind specific DNA sequences called S/MAR regions. These regions are involved in chromosomal replication, transcription, and interaction with topoisomerase II. NMPs have been investigated as potential cancer markers. The discovery that they are released into urine and blood suggested their role in cancer diagnosis [16].

Current research on the nuclear matrix protein NMP22 in the diagnosis of bladder cancer caused the NMP22 BladderChek test to obtain the FDA recommendation as a bedside “point-of-care” test for the rapid detection of cancer in risk groups in combination with cystoscopy. Its advantage is that positive results, expressed with the colorful immunodiffusion reaction, are already obtained after 30 min and require only a few drops of urine. This test is 6-fold cheaper than cytology. The disadvantage of the test is the possibility of false positive results due to the release of the protein NMP22 from dead cells, forming as a result of infection in the urinary system [34–36]. However, many researchers underline the higher diagnostic value of the NMP22 test than cytology particularly for the T stage and G grade. They propose the use of NMP22 determination to reduce the frequency of cystoscopy in monitoring low-risk groups (Ta, G1) [1, 34, 37, 38].

BLCA-4 was identified by Getzenberg et al. in 1996. It belongs to six bladder-specific nuclear proteins (BLCA-1–BLCA-6) expressed by bladder cancer cells. BLCA-4 interacts with several known transcription factors, such as AP-1 (activator protein-1), AP-2 (activator protein-2), NFATc (nuclear factor of activated T-cells), NF-E1 (nuclear factor erythroid 1), and NF-E2 (nuclear factor erythroid 2). These proteins may not effect proangiogenic pathways in bladder cancer; they can however interact with IL-1*α*, IL-8, VEGF (vascular-endothelial growth factor), and MMP-9 (matrix metalloproteinase-9) to enhance tumorigenesis and tumor invasiveness. The mechanism of BLCA-4 is poorly understood [14–16, 39].

The analysis of nuclear matrix protein in BC patients showed that the level of NMP22, BLCA-4, and NMBL in urine is significantly higher (*p* ≤ 0.001 in all) in BC than in healthy controls. The comparison of nuclear matrix proteins levels between T BC groups and between G BC groups shows some differences. The results are clear for isoenzyme GST*π* (Figure 1(b)). The invasiveness of tumor (T2) and higher grade (G2/G3) result in a higher level of GST*π*. Also, the BLCA-4 level increases with higher grade G (Figure 1(a)), and 8-OHdG increases with the invasiveness T (Figure 1(c)) except for TIS, but the TIS group is not representative because of the small number of cases (*n* = 7). In general, the experiments concerning the T and G BC group are limited by the small number of cases so the final conclusion is not proposed. However, the preliminary observation suggests the continuation of the investigation.

In search of a marker panel for diagnostic significance, which also takes into account exposure to xenobiotics and chemical carcinogenesis, we studied the level of nuclear matrix proteins in patients with slow acetylation genotype (SA) and with an increased degree of DNA damage expressed by 8-OHdG concentration. There were no significant differences in the values of NMP22 depending on the acetylation status, but a positive correlation was observed for NMP22 and 8-OHdG (*p* = 0.02). This correlation was significantly higher for the BLCA-4 protein in the slow acetylator group (*p* ≤ 0.001) than for IA and FA groups (Table 6). This indicates the highest diagnostic value of BLCA-4 protein, in the environmental exposure measured by the increase of 8-OHdG in urine. Numerous data confirm the value of 8-OHdG as a biomarker in exposure to xenobiotics. The examinations of inhabitants from the area of increased exposure to arsenic showed a significantly higher 8-OHdG level in the urine as the effect of chronic oxidative stress and DNA damage caused by arsenic compounds [40, 41]. A correlation between the concentration of arsenic in the blood and the level of 8-OHdG in the urine was found [26]. Also, an influence of other xenobiotics on the increase of 8-OHdG concentration in blood or urine was described [28, 42]. Some papers also describe the impact of smoking or exposure to carcinogens contained in tobacco smoke on the increase in 8-OHdG concentration [27]. Our own examinations confirmed this relation (*p* = 0.003). In recent years, there have also been a few reports noting the value of 8-OHdG as a prognostic marker in bladder cancer [43, 44]. The first investigation about the importance of 8-OHdG in BC was published in 2003 and showed that smokers with BC have higher (*p* < 0,001) 8-OHdG level in peripheral leukocytes than healthy smokers. This is in accordance with other observations about the increased level of 8-OHdG in cancerous tissues especially in age-related cancer [45]. However, the examination of 8-OHdG level in sera of 40 BC patients did not show any difference between control groups [46]. Also, urinary 8-OHdG is considered as a biomarker in cancer [47]. In our research, urinary 8-OHdG is used as a marker of environmental exposure (smoking) on the base of reports that 8-OHdG is increased in healthy smokers in comparison to healthy nonsmokers [27]. The obtained results that 8-OHdG level is significantly increased in BC-SA smokers in comparison to BC-SA nonsmokers confirmed our hypothesis. So, the correlation between nuclear matrix proteins and 8-OHdG seems fundamental for reaching conclusions.

Nuclear matrix protein BLCA-4 is of interest as a tumor-specific bladder cancer marker [14, 48–52]. Our experiments also suggest its importance in chemical carcinogenesis due to the high BLCA-4 with 8-OHdG correlation (*p* ≤ 0.001, Table 6). In our experiments, smoking was the marker of chemical carcinogenesis and environmental risk, especially interesting in BC SA group (sensitive for xenobiotics). Detailed evaluation of nuclear matrix proteins and other parameters was made in BC SA smokers group. The analysis of BC SA smokers showed the highest correlation value with 8-OHdG for BLCA-4 (*R* = 0.6942; *p* = 0.0001) (Table 6). This shows that, among all examined nuclear matrix proteins, BLCA-4 is the best marker which reflects the environmental risk of chemical cancerogenesis. The BLCA-4 correlated also with GST*π* activity in the urine (*p* = 0.017) and tumor stage T (*p* = 0.005) (Table 6). Examinations of animals with bladder cancer showed that the expression of this protein had appeared already in the eighth week after initiation of the cancerous process, that is, 22 weeks more quickly than characteristic cancerous changes.

Our study indicates the higher diagnostic value of the total protein pool of the nuclear matrix (NMBL) than the single protein, NMP22 or BLCA-4, in bladder cancer. Interesting results were obtained for NMBL in the BC SA group. NMBL showed positive correlations with GST*π* and 8-OHdG (Table 6). Moreover, the NMBL in BC group had higher values of estimated statistical factors (NPV, LR(+), and AUC) in comparison with NMP22 and BLCA-4. In SA group, specificity and PPV reached 100%. The rest of the factors such as LR(−) and AUC were also higher in the SA group than in total BC patients (Table 7). The statistical analysis using by Wald's and Hosmer's tests indicated NMBL as an important parameter in the assessment of the likelihood of the disease.

Many data show that cigarette smoking, and therefore exposure to carcinogens contained in tobacco smoke, is an important risk factor for BC [2–4]. Among many other environmental risk factors, this addiction occupies the first place as the cause of BC. There is also evidence of the importance of genetic predisposition to BC, especially the slow acetylation genotype NAT2 [5, 6, 53]. Several studies also point to 8-OHdG and GST*π* as markers of exposure and the ability of detoxification of the body [21, 22, 26–28]. The significant contribution of exposure to carcinogens to the development of BC necessitates the search for markers that reflect individual sensitivity to environmental toxins. Our study suggests the utility of BLCA-4 due to its strong correlation with 8-OHdG, in particular among the slow acetylator group SA. Another marker highly correlated with 8-OHdG, especially in the SA group, is the isoenzyme GST*π*.

## 5. Conclusions

The total pool of nuclear matrix proteins in the urine (NMBL) has a higher diagnostic value in BC than single proteins NMP22 and BLCA-4. Among all examined nuclear matrix proteins, BLCA-4 is the best marker which reflects the environmental risk of chemical cancerogenesis, among other parameters GST*π*.

## Figures and Tables

**Table 1:** Demographic and clinical data for the BC patients and the control.

Patients	
Total number (*n*)	91
Male/female	74/17
Age (years) (mean ± SD)	68 ± 9.5
Smokers number	64 (75.3%)
Clinical grading	
Ta	43 (45.3%)
T1	31 (34.1%)
T2	10 (11%)
TIS	7 (7.7%)
Clinical staging	
G1	40 (47.6%)
G2	41 (48.8%)
G3	3 (3.6%)
Controls total number (*n*)	25
Male/female	18/7
Age (years) (mean ± SD)	65 ± 6.9
Smokers number	19 (76%)

**Table 2:** Results from the questionnaire of patients with BC.

Factor risk of BC	Patients with BC (*n* = 85) [%/*n*]
Smoker patients	75.4% (*n* = 64)
Living in the city	71% (*n* = 58)
Contact with pesticides	34.1% (*n* = 21)
Exposure to chemicals (living near factories)	13.4% (*n* = 11)

**Table 3:** Mean values of studied parameters in examined groups.

	NMP22	BLCA-4	NMBL	GST*π*	8-OHdG
	[ng/mg cr.]	[ng/mg cr.]	[ng/mg cr.]	[ng/mg cr.]	[ng/mg cr.]
*BC*					
$\overset{-}{x}$	10.846^*∗*^	3.015^*∗*^	14.454^*∗*^	22.545^*∗*^	18.914^*∗*^
SD	8.312	3.535	11.919	29.878	15.468
*n*	91	63	63	91	63

*C*					
$\overset{-}{x}$	5.147	0.878	6.026	4.210	12.393
SD	2.025	0.477	2.305	3.429	3.682
*n*	25	25	25	25	25

*Females (BC)*					
$\overset{-}{x}$	10.746	3.691	18.750	28.517	21.577
SD	8.205	5.295	22.076	29.554	20.322
*n*	17	10	10	17	10

*Males (BC)*					
$\overset{-}{x}$	10.285	2.887	13.655	21.174	18.441
SD	6.689	3.153	8.990	25.816	14.559
*n*	74	53	53	74	53

*Smokers (BC)*					
$\overset{-}{x}$	11.054	3.500	15.519	23.230	21.486^■^
SD	9.551	4.027	13.819	31.648	17.538
*n*	64	43	43	64	43

*Nonsmokers (BC)*					
$\overset{-}{x}$	10.565	2.337	12.825	24.292	14.340
SD	3.987	2.032	6.119	28.205	8.486
*n*	21	14	14	21	14

*∗*: statistically significant difference in comparison to control group; ■: between smokers and nonsmokers; $\overset{-}{x}$: mean; SD: standard deviation; *n*: number of cases.

**Table 4:** Analysis of genotype and acetylation status in BC patients.

Genotype	Acetylation status	*n* (%)
*NAT2*		
^*∗*^4/^*∗*^4	Fast	8 (8.8)
^*∗*^4/^*∗*^5	Fast	11 (12.1)
^*∗*^4/^*∗*^6	Intermediate	17 (18.7)
^*∗*^4/^*∗*^7	Intermediate	1 (1.0)
^*∗*^5/^*∗*^5	Slow	16 (17.6)
^*∗*^5/^*∗*^6	Slow	26 (28.6)
^*∗*^5/^*∗*^7	Slow	3 (3.3)
^*∗*^6/^*∗*^6	Slow	4 (4.4)
^*∗*^6/^*∗*^7	Slow	5 (5.5)

**Table 5:** Mean value of markers at various NAT2 acetylators.

NAT2 acetylation status	NMP22 [ng/mg cr.]	BLCA-4 [ng/mg cr.]	NMBL [ng/mg cr.]	GST*π* [ng/mg cr.]	8-OHdG [ng/mg cr.]
*SA*	*Slow*				
$\overset{-}{x}$	11.757	3.087	15.097	23.542	19.905
SD	9.257	3.448	13.154	32.732	15.497
*n*	54	42	42	54	42

*IA*	*Intermediate*				
$\overset{-}{x}$	10.093	3.011	14.328	23.319	16.971
SD	7.265	4.194	9.812	27.805	17.032
*n*	29	17	17	29	17

*FA*	*Fast*				
$\overset{-}{x}$	7.425	2.272	8.390	13.016	16.911
SD	2.517	1.106	1.660	12.627	9.522
*n*	8	4	4	8	4

**Table 6:** The correlations between examined parameters in the group of slow acetylators (SA).

Correlations in SA group	*R*	*p*
BLCA-4/GST*π*	*R* = 0.3656	*p* = 0.0172
BLCA-4/T (*stage*)	*R* = 0.4224	*p* = 0.0053
NMBL/GST*π*	*R* = 0.3154	*p* = 0.0418
NMBL/T (*stage*)	*R* = 0.3782	*p* = 0.0135
GST*π*/G (*grade*)	*R* = 0.4550	*p* = 0.0005
GST*π*/T (*stage*)	*R* = 0.3715	*p* = 0.0056
8-OHdG/smoking	*R* = 0.3782	*p* = 0.0192
8-OHdG/T (*stage*)	*R* = 0.3549	*p* = 0.0210
8-OHdG/NMP22	*R* = 0.3631	*p* = 0.0180
**8-OHdG/BLCA-4**	**R** = 0.5834	**p** = 0.0001
8-OHdG/NMBL	*R* = 0.3941	*p* = 0.0098
8-OHdG/GST*π*	*R* = 0.3941	*p* = 0.0098
**8-OHdG/BLCA-4** ^*∗*^	**R** = 0.6942	**p** = 0.0001
8-OHdG/NMBL^*∗*^	*R* = 0.6053	*p* = 0.0008
BLCA-4/GST*π*^*∗*^	*R* = 0.4332	*p* = 0.0185
NMBL/GST*π*^*∗*^	*R* = 0.3762	*p* = 0.0441
8-OHdG/NMP22^*∗*^	*R* = 0.4541	*p* = 0.0131
8-OHdG/GST*π*^*∗*^	*R* = 0.4201	*p* = 0.0234

SA: slow acetylation; *R*: Spearman correlation coefficient; *p*: level of significance; *∗*: correlation in smokers SA group.

**Table 7:** Evaluated indicators for assessing the reliability of diagnostic tests used in analysis (measures of association and measure of effect) in the whole BC and SA subgroups.

Index	NMP22		BLCA-4		NMBL		GST*π*		8-OHdG	
	BC	SA	BC	SA	BC	SA	BC	SA	BC	SA
Sensitivity [%]	57	71	65	69	64	57	81	91	32	55
Specificity [%]	96	80	88	88	96	100	72	72	100	84
ACC [%]	66	75	72	76	73	74	79	84	51	66
PPV [%]	98	86	93	91	98	100	91	85	100	82
NPV [%]	38	63	50	63	51	58	51	82	37	53
LR(+)	14.286	3.571	5.423	5.754	15.873	—	2.904	3.231	—	3.423
LR(−)	0.446	0.357	0.397	0.352	0.380	0.429	0.259	0.132	0.683	0.539
AUC	0.836	0.818	0.830	0.835	0.858	0.862	0.790	0.866	0.637	0.706
Youden index	0.53	0.56	0.53	0.57	0.59	0.60	0.53	0.62	0.32	0.39
Cut-off point	8.53	8.529	1.403	1.4	9.740	10.45	4.342	4.38	18.17	15.322

ACC: accuracy; PPV: positive predictive value; NPV: negative predictive value; LR(+): positive likelihood ratio; (LR−): negative likelihood ratio; AUC: area under the ROC curve; Youden index (0-1) showing the relationship between sensitivity and specificity tests.
